# Pulmonary Artery Dissection Post-blunt Thoracoabdominal Trauma

**DOI:** 10.5811/cpcem.2019.12.44649

**Published:** 2020-06-02

**Authors:** Daniel Quesada, Larissa A. Morsky, Amber Jones, Allan L. Capote

**Affiliations:** * Kern Medical, Department of Emergency Medicine, Bakersfield, California; † Kern Medical, Department of Surgery, Bakersfield, California; ‡ LAC+USC Medical Center, Department of Emergency Medicine, Los Angeles, California

**Keywords:** pulmonary artery dissection, blunt thoracic trauma

## Abstract

**Background:**

Pulmonary artery dissection is a rare condition that is usually diagnosed in patients exhibiting chronic pulmonary arterial hypertension, congenital heart abnormalities or secondary to iatrogenic injury. Diagnosis is often made at autopsy as many patients experience sudden death when the pulmonary artery dissection progresses rapidly and ruptures into the pericardium, resulting in acute cardiac tamponade.

**Case Presentation:**

We report a case of pulmonary artery dissection, which resulted from blunt thoracic trauma diagnosed in the emergency department.

## CASE PRESENTATION

**Image 1:**
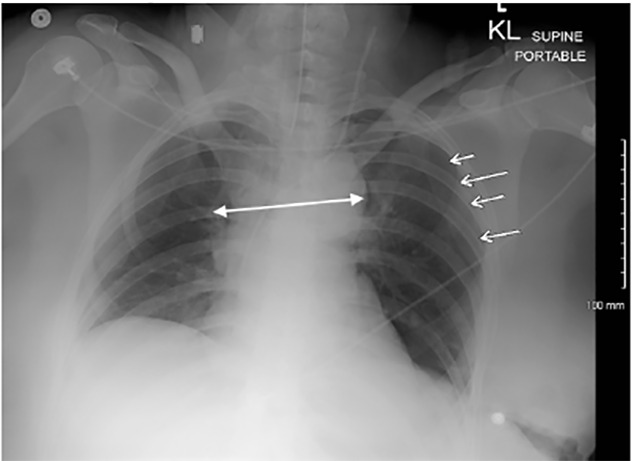
Anteroposterior supine chest radiograph revealing slight mediastinal widening (double-headed arrow), pneumothorax (arrows), and multiple left-sided rib fractures.

**Image 2:**
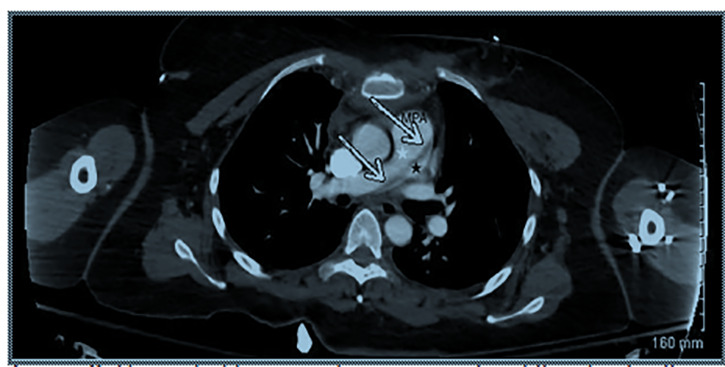
Computed tomography angiography of the chest with intravenous contrast in axial view showing displacement of the left atrium (arrows). True (white star) and false (black star) lumens can also be visualized.

A 43-year-old restrained female with an unremarkable past medical history was involved in a frontal impact high-speed motor vehicle accident and presented to our emergency department with a deceleration injury. Upon arrival, she was alert and fully oriented with vital signs within normal limits. She reported vehicle airbag deployment, loss of consciousness on impact and noted sternal and abdominal pain. On physical examination, secondary trauma survey was positive for diffuse sternal and abdominal tenderness with a large seatbelt sign across the chest and abdomen. Focused assessment with sonography for trauma was positive in Morrison’s pouch, consistent with significant thoracoabdominal trauma. A chest radiograph revealed mediastinal widening (Image 1) and a left-sided pneumothorax. Given concern for intrathoracic injury, a computed tomography (CT) angiography of the chest was performed and displayed a post-traumatic pulmonary artery dissection (PAD) with classic findings of true and false lumens (Image 2). Other CT findings included Cervical(C) 5, C6 and C7 transverse process fractures and a linear laceration at the inferior and posterior portion of the right lobe of the liver with minimal amount of free fluid surrounding the liver.

Since the PAD was deemed stable, an exploratory laparotomy was performed for clinical as well as radiographic findings, revealing 3 areas of mesenteric avulsion resulting in small bowel resection and control of hemorrhage. A damage control procedure was done with temporary abdominal closure, remained intubated and was transferred to an outside facility where she was lost to follow-up.

## DISCUSSION

This report describes a case of traumatic PAD. Most reported traumatic injuries of the pulmonary artery have occurred secondary to blunt or penetrating chest trauma and result in rupture, pseudoaneurysm or both.^1^,^2^ The majority of patients with PAD are diagnosed post-mortem due to the condition manifesting as cardiogenic shock or sudden death when the dissection progresses rapidly and results in rupture.^3^ However, a recent review of the literature has noted over 90% of traumatic, non-iatrogenic pulmonary artery injuries of 50 reported since 1990 have resulted in survival of the patient.^2^ Diagnosis in living patients has been made based on intraoperative findings or pulmonary arteriography.^3^ Clinical suspicion for a PAD should include: chest pain, cyanosis, pulmonary arterial hypertension and dyspnea.^4^,^5^

**CPC-EM Capsule**

What do we already know about this clinical entity?

The majority of reported pulmonary artery dissections are diagnosed post-mortem. Most of the reported dissections rupture, causing cardiogenic shock or sudden death.

What is the major impact of the image(s)?

A case of traumatic pulmonary artery dissection diagnosed in a living patient, displaying clear displacement of the left atrium.

How might this improve emergency medicine practice?

This case reminds emergency physicians to maintain pulmonary artery dissection in the differential when encountering high speed frontal impact deceleration injuries or other major traumatic injuries to the chest.

Although there is no consensus on management due to the variation of mechanism of injury and rarity of the condition, the mainstay of treatment for traumatic PADs is a surgical or interventional approach.^2^

## References

[1] Collins MP, Robinson GC (1989). Traumatic Rupture of the Pulmonary Artery. Ann Thorac Surg.

[2] Yanagawa Y, Ishikawa K, Nagasawa H (2018). Traumatic pulmonary artery injury: a review of the recent literature. Vessel Plus.

[3] Graham JK, Shehata B (2007). Sudden death due to dissecting pulmonary artery aneurysm: a case report and review of the literature. Am J Forensic Med Pathol.

[4] Jung LY (2016). Is pulmonary artery dissection predictable?. Heart Lung.

[5] Khattar RS, Fox DJ, Alty JE (2005). Pulmonary artery dissection: an emerging cardiovascular complication in surviving patients with chronic pulmonary hypertension. Heart.

